# Design and Performance of a Neurosurgery Assisting Device

**DOI:** 10.3390/biomimetics10060345

**Published:** 2025-05-23

**Authors:** Karla Nayeli Silva-Garcés, Marco Ceccarelli, Matteo Russo, Christopher René Torres-SanMiguel

**Affiliations:** 1 Instituto Politécnico Nacional, Escuela Superior de Ingeniería Mecánica y Eléctrica, Sección de Estudios de Posgrado e Investigación, Unidad Zacatenco, Mexico City 00738, Mexico; ksilvag1000@alumno.ipn.mx; 2 LARM2 Laboratory of Robot Mechatronics, University of Rome Tor Vergata, 00133 Rome, Italy; marco.ceccarelli@uniroma2.it (M.C.); matteo.russo@uniroma2.it (M.R.)

**Keywords:** neurosurgery, assisting devices, parallel manipulators, design, performance evaluation

## Abstract

This paper presents a new design solution for a neurosurgery-assisting device (NeurADe) based on a 3-RPS parallel kinematic mechanism. The NeurADe design employs compact linear actuators to accurately insert a cannula into specific areas of the brain. The CAD design and assembly of a prototype are discussed in this paper. The preliminary NeurADe prototype features 3D printed parts and incorporates mechanical and electrical components, which are designed for ease of use and lightweight functionality. For design validation and operational characterization, sensors measuring current, acceleration, and force data were utilized, and testing results are discussed to prove the feasibility of the proposed design.

## 1. Introduction

The development of surgery platforms that integrate robotic solutions marks a significant evolution, introducing modular designs, haptic feedback, reduced invasiveness, and enhanced portability [1]. Robotic surgical technology has undergone a rapid extension over the last 20 years with differentiations according to its clinical applications in diverse designs: soft-tissue robotic platforms, orthopedic robotic platforms, endoluminal robotic platforms, neurosurgery, and spine platforms [2]. In the neurosurgical field, modern robotic surgical systems are equipped with highly dexterous arms, and miniaturized instruments reduce surgeon arm tremors and enable delicate maneuvers. The implementation of advanced materials and designs, along with the integration of imaging and visualization technologies, have enhanced surgical accuracy and made robots safe and adaptable to various procedures [3]. Thirteen studies, with a total of 538 robot-assisted neuro-interventions, were examined in [4], in which the use of a robotic platform in neurosurgery was concluded to be highly feasible, safe, and capable of performing specific tasks. These platforms also provide efficiently integrable, expandable, and low-cost systems for neurosurgery, which currently uses stereotactic frames. In the future, semi-independent artificial intelligence-assisted robotic operations will be performed by artificial intelligence for limited surgical interventions [4].

Human-based surgery is a complex and challenging process that needs to be extrapolated into robotic-based surgery. Surgeons have a subjective point of view, whereas robots can be entirely objective. The application of robots in neurosurgeries is still limited due to the anatomical difficulty of differentiating the tissues and the possibility of mechanical failure in autonomous robots [5]. The Mayfield headframe and Sugita do not have a gauge to monitor pressure. Scientists have started incorporating machine learning models to analyze tool–tissue interaction-force data in neurosurgery procedures for systematic feedback without obtaining specific results, as reported in [6]. The forces exerted during neurosurgical interventions have a range from 0.4 N to 14 N. The use of robotic platforms with force feedback mechanisms can improve surgical performance and decrease tissue damage, as discussed in [7].

Thus, robotic surgery is focused on solving neurosurgeons’ needs in the pre-, intra-, and postoperative stages of neurosurgery when working with limited displacements, high accuracy and small size, and when exerting sufficient force without necessarily imitating the same surgeon gestures to achieve the same task [8]. Future directions for robotics in neurosurgery have the objective of increasing automation in procedures such as deep brain stimulation and robot-assisted spinal surgery. The use of artificial intelligence (AI) in applications of surgical instruments will enhance surgical precision, training, and outcomes [9]. The review in [10] explains that in 19 neurosurgeries, the mean time for setting up some robot stereotaxis systems was 57.4 ± 10.7 min, and the mean radial errors of trajectory relative to the planned trajectory at the entry and target points were 0.625 ± 0.443 mm and 0.745 ± 0.472 mm, respectively. The review in [11] explores the implementation of AI and robotic technologies in surgical practices in some medical fields in 2024. The findings indicate that the use of AI-driven surgical systems improves decision-making and reduces surgical errors. Parallel kinematic mechanisms are offering more innovations in the neurosurgery-assisting device field than their serial counterparts, including greater rigidity, load capacity, precision, and speed [12]. Neuromate, Neuroarm, Neurobot, NaoTract, Remebot, and ROSA are examples of commercial devices that are widely used nowadays. Neuromate is a five-joint and six-degree of freedom (DoF) manipulator, which is affected by errors of less than 2 mm [13,14]. Neuroarm is a 7-DoF manipulator with shoulder, elbow, and wrist joints, capable of holding a maximum payload of 750 g at 200 mm/s. Neurobot is a 3-DoF manipulator and has the capacity to transport equipment with a 1 mm diameter. ROSA is equipped with sensitive 6-DoF manipulators for manipulating instruments [15]. The lightweight, table-mounted robotic alignment tool in [16] was used in 26 surgical procedures with target errors of 1.4 ± 1.2 mm and 2.6 to 1.6 mm. Moreover, the work in [17] determined multidimensional motion capabilities of both platform prototypes with a motion positioning resolution reaching a level of 0.1 μm. The work in [18] presents a parallel robot for minimally invasive surgery that has a modular architecture of a spherical parallel mechanism with an RCM and a 4-DOF parallel robot that actuates the surgical instrument.

When designing a neurosurgery-assisting device, the application scenarios and environmental information should be fully considered, and the design should be based on the characteristics of the human body’s structure. In [19], a design methodology for developing a custom-made optomechanical end-effector design is presented for testing in combination with a high-precision robotic system capable of providing sub-millimeter motion accuracy. The robot in [20] is a 7-DOF, MRI-guided stereotactic neurosurgical robotic system. For the preoperative trajectory, a 5-DOF and a 2-DOF are used to determine the depth and twisting motion of the needle during insertion. The video-based platforms give promising results, allowing AI-driven assessment with the use of several measurements and providing relevant feedback based on previous and contemporary data [21]. The uses of parallel robots, joint motion, and linear motion provide ideal solutions for targeting the position from its home/safe position to move the end-effector to the final desired position [22]. The NeurADe solution proposed in [23] is an example of the application of parallel kinematic mechanisms.

This paper presents the design and prototype of a novel design solution for a neurosurgery-assisting device, NeurADe, as an evolution of the architecture that is introduced in [24,25]. The prototype has been assembled and tested within predefined test modes to validate the design. The results are presented in terms of acceleration, current of the device, and force applied in the screws and at the cannula.

## 2. Materials and Methods

This section presents the requirements for designing a neurosurgery-assisting device with a parallel mechanism for trajectory planning for electrode insertion. The proposed version is based on a parallel mechanism with an anthropometric design that is tailored for the adult head.

### 2.1. Requirements

**Figure 1:**
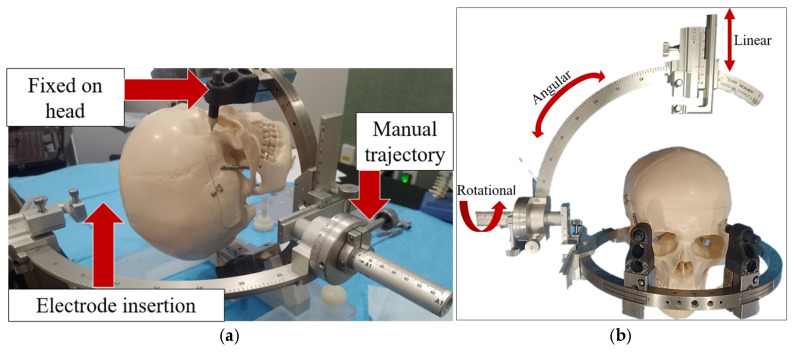
Market neurosurgery-assisting device, [3]: (**a**) components and (**b**) degrees of freedom.

Neurosurgery for electrode insertion has three main procedures: pre-operation, targeting, and operation. During the pre-operation, the surgeon collocates a stereotactic frame in the patient′s head [2]. The frame is a neurosurgery-assisting device capable of targeting a specific point in the brain when positioned on the patient′s head. Figure 1 shows a market neurosurgery-assisting device.

Figure 1a shows the main components of a market neurosurgery-assisting device. The device is fixed to the head using special anesthesia and four screws: two screws in the frontal bone and two screws in the parietal bone [3]. During the targeting process, the patient is transferred to an area where a computer tomography (CT) image is generated. This allows for the precise localization of the targeted area in the brain and visualization of the coordinates. At the start of the surgery, the surgeon secures a stereotactic frame to the patient′s head. The procedure is completed with the electrode insertion. Market neurosurgery-assisting devices have between three and four degrees of freedom and between one and two end-effectors, as shown in Figure 1b.

**Figure 2:**
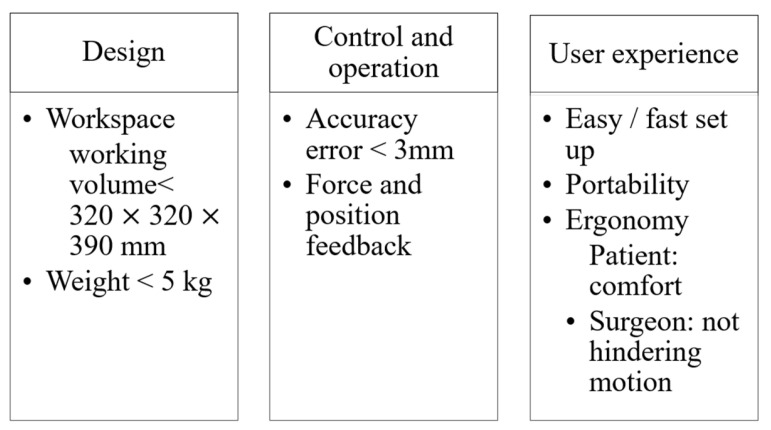
Design requirements for a neurosurgery-assisting device.

The rotational component of the system is designed to position it near the parietal lobe. This area contains the soft tissues that produce stimulation. The angular part of the system is responsible for placing the electrode cannula mechanism in either the right or left parietal lobe. The linear section is used to insert the cannula that holds the electrodes. Neurosurgery-assisting devices for electrode insertion applications have to fulfill medical requirements, such as being designed with metallics or polymeric biomaterials. The devices need to be sterilizable or designed as a single-use device. Its design should be focused on its use in CT or MR imaging [4]. The technical requirements for a neurosurgery-assisting device that uses a frame mounted on the head of the patient are summarized in Figure 2.

For the design requirements, the workspace of the complete device has to fulfill a working volume corresponding to the size of the head with maximum dimensions of 320 mm in length, 320 mm in width, and 390 mm in height. The design should not exceed a total weight of 5 kg. For the controlled operation of the device, it is essential to consider an accuracy error of less than 3 mm, as well as feedback on the applied force to secure the device position. Because surgeons will be using the device, the user experience should prioritize easy and quick setup and portability. Regarding ergonomics, the design should be focused on patient comfort without impeding its easy use by surgeons.

### 2.2. Conceptual Design

**Figure 3:**
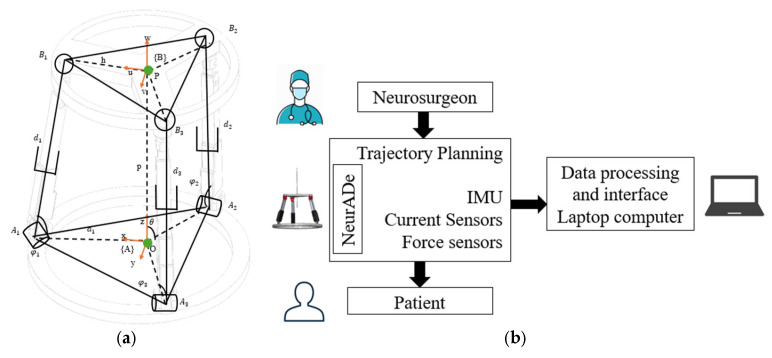
Conceptual design of NeurADe: (**a**) kinematic model and (**b**) a scheme of the device design.

**Figure 4:**
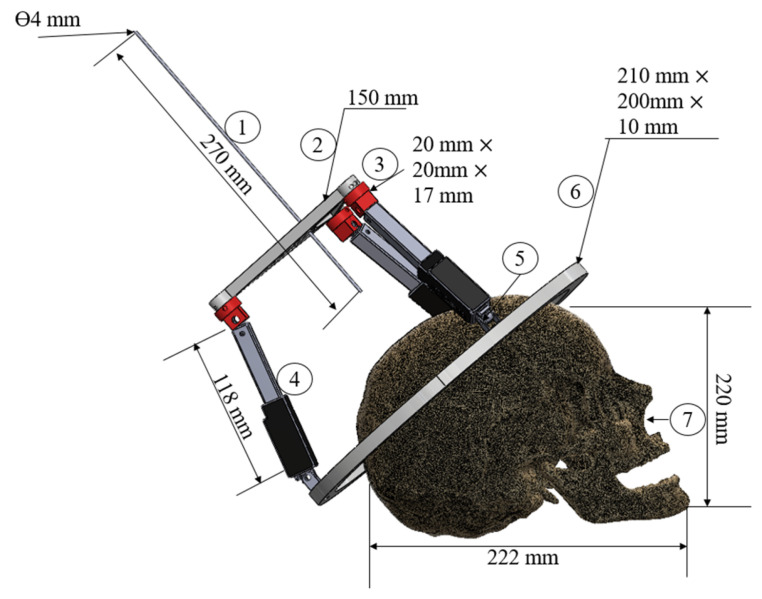
CAD design of NeurADe: a CAD design (1: cannula, 2: mobile base, 3: spherical joints, 4: linear actuators, 5: revolute joints, 6: fixed base, 7: cranium mockup).

Figure 3 shows the conceptual design of the NeurADe. Figure 3a presents the kinematic model of the device, which is a 3-RPS parallel mechanism and has a fixed oval base to be placed around the head. The device has three linear actuators with revolute and spherical joints at their proximal and distal ends, respectively. At the top of the device, there is a mobile platform with a cannula in its center. The motion parameters of the device are the pitch, roll, and yaw angles that define the orientation of the mobile platform. Figure 4 is a scheme of the device’s design. The operator or neurosurgeon uses the NeurADe to implant it in the head of the patient or user. Linear actuators power the device, while data are gathered using an inertial measurement unit (IMU) together with current and force sensors. These data are processed on a laptop computer, which also includes a user interface that enables the operator or neurosurgeon to adjust the trajectory planning.

The preliminary prototypes of the NeurADe [25] encountered issues during laboratory tests. Specifically, problems arose when the device was mounted on the cranium mockup. The base of the device was not securely attached to the cranium, leading to instability. Additionally, the force required to tighten the screws provided no feedback, complicating the adjustment process. Another issue was generated by the oversized linear actuators that had strokes of 100 mm, which contributed to trajectory errors and decreased stiffness when the cannula was elevated too much during the insertion into the brain. To address these issues, the 3-RPS platform will require a reduction in the size of the linear actuators to minimize trajectory errors. In the prototype presented here, the linear actuators are reduced and have a maximum stroke of 50 mm to improve their performance and accuracy.

### 2.3. CAD Design and Modeling

Figure 4 presents the CAD design elaborated in Solidworks 2023^®^ for performance simulation. The device has seven principal components, as illustrated in the figure.

The cannula is the device used for insertion when placing electrodes during neurosurgery [3]. The linear mechanism has a cannula with a diameter of 4 mm and a length of 270 mm. The cannula is a round tube of aluminum or steel. The cannula extends from the top of the mobile base towards the designated area required by the commercial skull. An inertial measurement unit (IMU) model BMI160 [26] is embedded in the mobile base alongside the cannula. This sensor collects data on the motion performance of the devices, measuring accelerations as well as pitch, roll, and yaw angles. The mobile base is an equilateral triangle of 150 mm on each side that is designed to support the cannula, the IMU, and the spherical joints. The spherical joints are obtained by using universal joints with rotating axes that are attached to the corners of the mobile base. The overall dimensions of the prototype are 220 mm × 17 mm in total length, and it is made of steel. An additional component is located between the spherical joint and the linear actuator to enable proper movement. This component is a Thermoplastic polyurethane TPU part that is created by a 3D printer and has a diameter of 15 mm and a height of 10 mm. Three Actuonix L16-50-63-R [27] are used as linear actuators. The actuator has 50 mm of maximum stroke and acts as a linear servomotor. The revolute joints are 10 mm × 5 mm × 10 mm and, as fixturing components, are compatible with the Actuonix servomotor (Actuonix motion devices Inc. ®, Rome, Italy). They were selected for proper attachment and performance between the linear actuators and the fixed base, and they are made of aluminum alloys. The fixed base has an oval shape that was 3D printed in ABS material and is designed to fit comfortably around the patient′s head, reflecting the dimensions of a human head. Its size is 220 mm × 200 mm × 10 mm, with three connections between linear actuators and screws. The fixed base uses four screws to fix the base to the cranium. The base has four commercial screws that are composed of M8 × 50 mm stainless steel DIN603. The cranium is an essential component of the NeurADe setup to simulate neurosurgery scenarios for patients. The commercial skull used for testing is based on adult anthropometric measurements of 220 mm × 222 mm × 180 mm. It is constructed from non-toxic PVC material, which is widely used by medical students, educators, and dentists and was developed with 3D-printed materials in TPU and ABS. Although these materials have been used in various medical devices, and the prototype may be considered as a single-use [28,29,30], the prototype was developed without applying regulations for sterility. The future prototype will be made of biocompatible materials such as 316L steel and PEEK.

## 3. Results

### 3.1. Prototype Assembly

**Figure 5:**
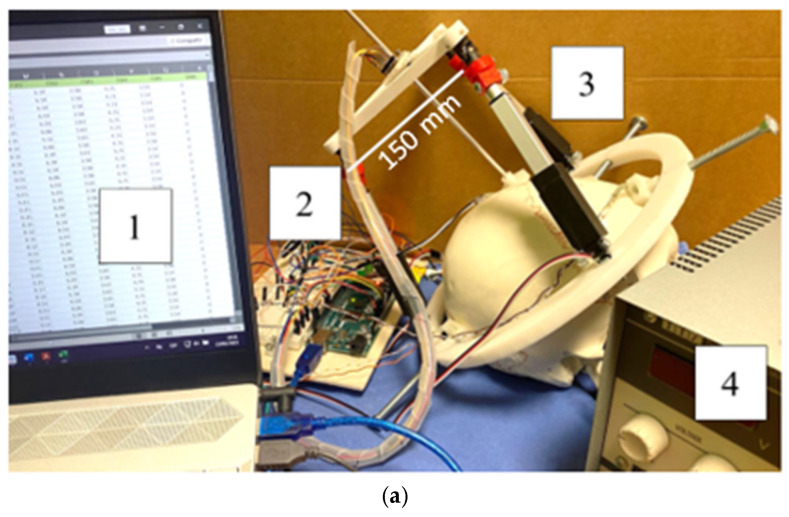

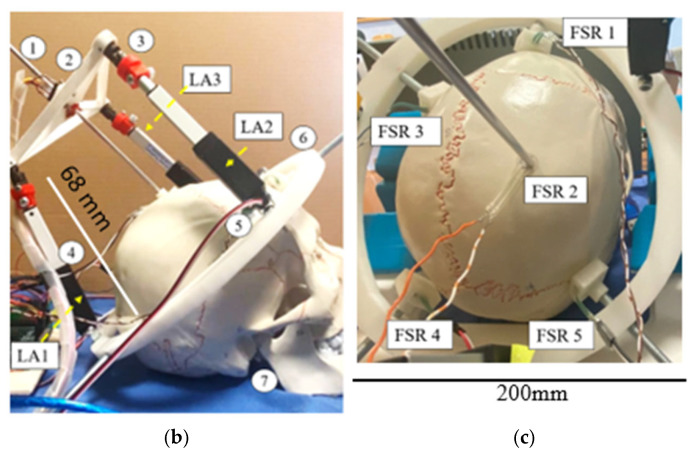
Prototype of NeurADe: (**a**) components (1: computer, 2: electric circuit, 3: prototype, 4: power supply), (**b**) assembly (1: cannula, 2: mobile base, 3: spherical joint, 4: linear actuator, 5: fixed base, 6: screw, 7: commercial cranium), and (**c**) upper view of the assembly.

Figure 5 describes the prototype of the NeurADe and presents the components, such as the laptop computer for data analysis, the electric circuit, the prototype set in the cranium, and the external power supply of 6 V for linear actuator activation.

**Figure 6:**
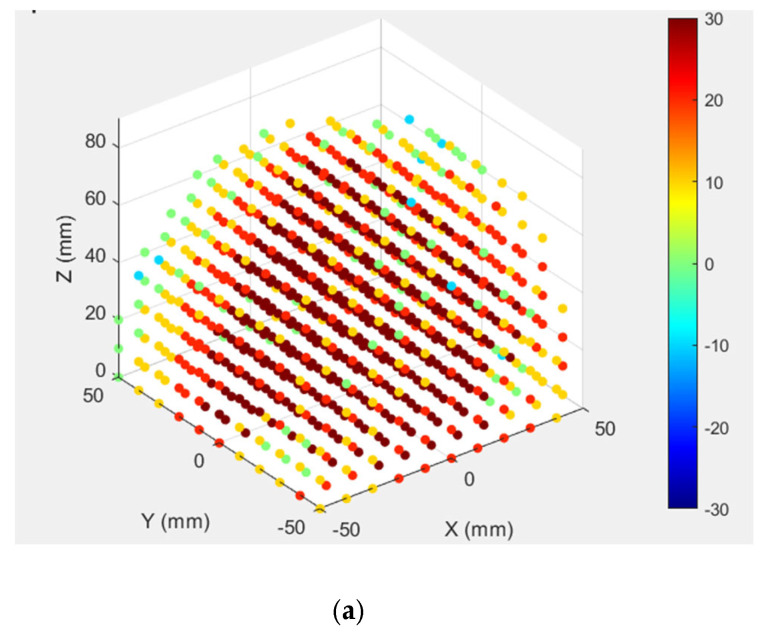

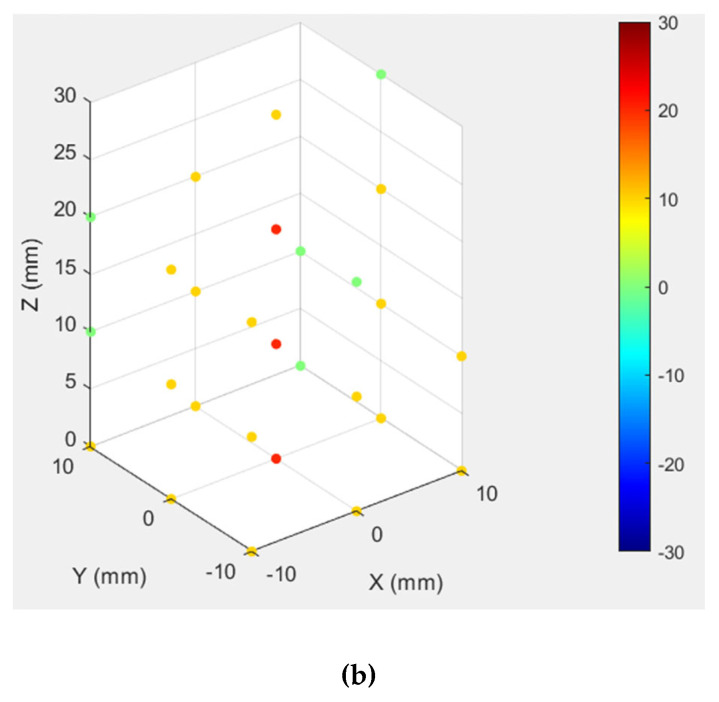
Workspace of NeurADe: (**a**) with a maximum stroke of 100 mm and (**b**) with a maximum stroke of 50 mm (color dots and colorbar represents theta values of workspace).

Figure 5b shows the assembly of the NeurADe, the components of which are presented in Figure 5. The linear actuators (LA) were connected between fixed and mobile bases. Figure 6 presents an upper view of the assembly. For the force feedback of the device, five Force Sensitive Resistors (FSR sensors) were used [31]. Four FSR sensors were connected between the screw and the cranium. The FSR2 was attached at the end of the cannula and the cranium. Figure 6 presents the workspace calculated on Matlab^®^ 2024 version. Figure 6a shows the workspace when the linear actuators were selected with a maximum stroke of 100 mm. Figure 6b presents the workspace of the linear actuators that had a maximum stroke of 50 mm. Figure 6b shows a minimization of the workspace compared with Figure 6a; the reduction of the workspace is necessary for the application of inserting the electrode into STN or GPi.

**Figure 7:**
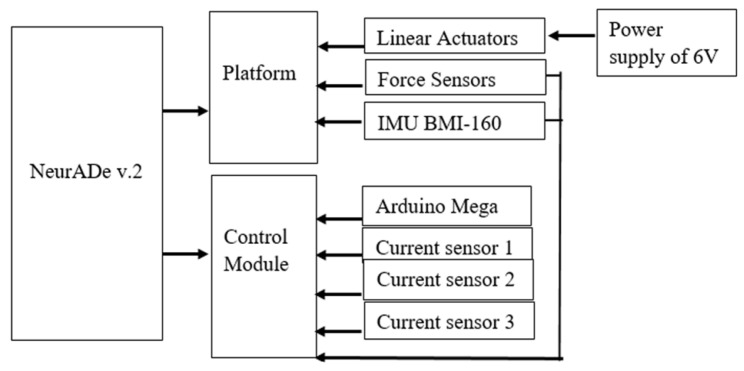
Scheme of control architecture of NeurADe.

Figure 7 shows a scheme of an electric circuit of the device. The 3-RPS platform is assembled with three linear actuators, and the IMU is attached to the center of the mobile base. The total weight of the prototype is 494 grs. An external power supply of 6 V is used to power the linear actuators. The control module has an Arduino Mega [32]. The force sensors acquire data from the force applied to the screws when the fixed base is set in the cranium and the cannula. The Arduino Mega serves as the primary component for data processing and control. The control module includes three current sensors, ACS-712 5 A [33], that monitor the current flowing from the linear actuators. The inertial measurement unit (IMU) gathers data on acceleration along the x, y, and z axes. Once the data are processed, the pitch, yaw, and roll angles are displayed.

### 3.2. Testing Layout and Model

**Figure 8:**
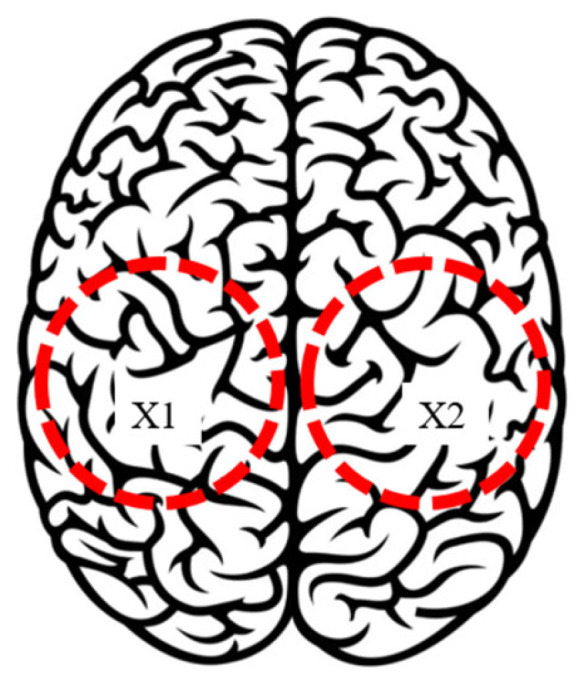
Scheme of input in test modes with NeurADe (Red circle in X1 represent the area of left parietal lobe and red circle in X2 represent the area of right parietal lobe).

The experimental test modes for the NeurADe are listed in Table 1. Two test modes were set to represent the device′s contact with the left and right parietal lobe zones, as illustrated in Figure 8. The output includes the acquisition data of accelerations, angles, current, power consumption, and force. For each test mode, three samples were acquired over 45 s, 75 s, and 90 s, the same as in the test in [20,21,22]. Data were acquired at a frequency of 50 Hz.

### 3.3. Test Results

**Figure 9:**
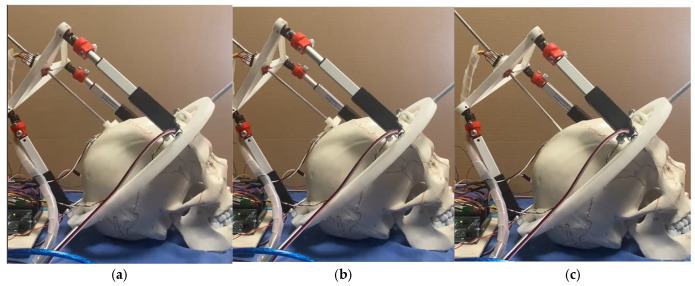
Performance of NeurADe in test mode 1: (**a**) start position, (**b**) performance to X1, and (**c**) final position.

Figure 9 shows a snapshot of test mode 1. The device starts with actuators LA3 and LA2 at a 10 mm stroke. It then moves these actuators to a 25 mm stroke and finally to a 0 mm stroke.

**Figure 10:**
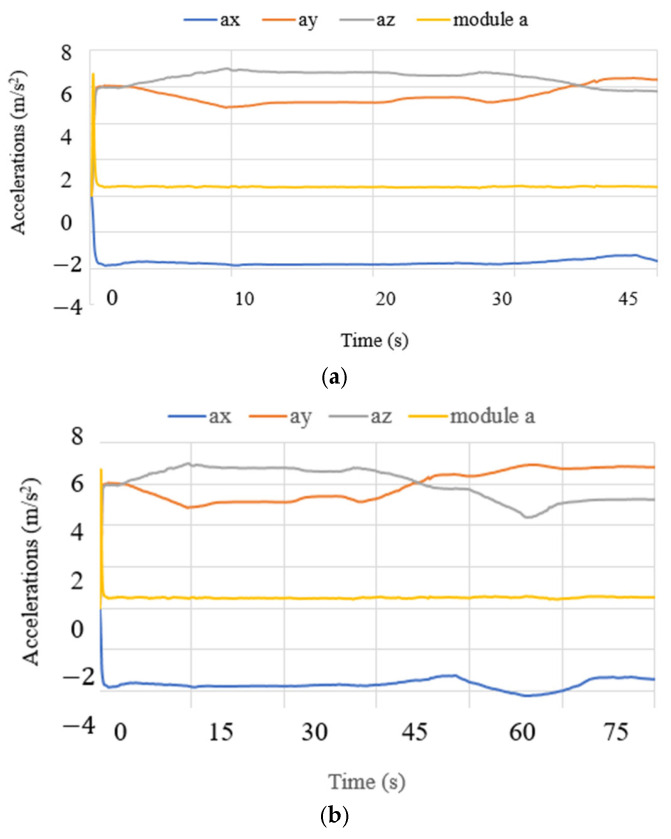

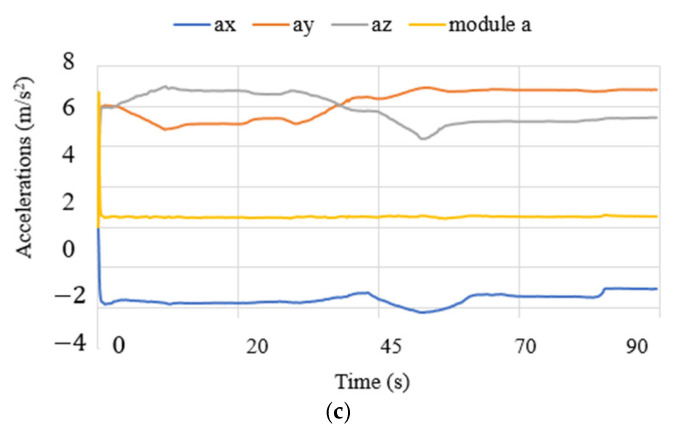
Acceleration acquired from test mode 1 with the NeurADe model from Figure 9: (**a**) 45 s, (**b**) 75 s, and (**c**) 90 s.

**Figure 11:**
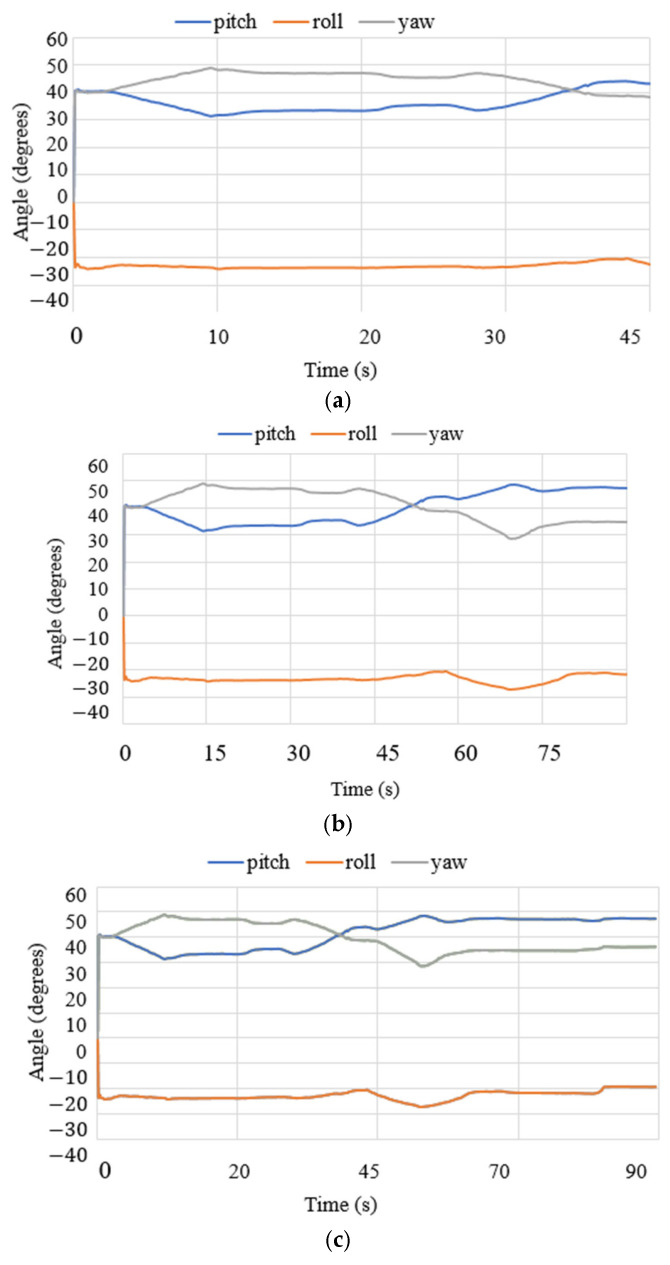
Angles acquired from test mode 1 with the NeurADe model from Figure 9: (**a**) 45 s, (**b**) 75 s, and (**c**) 90 s.

**Figure 12:**
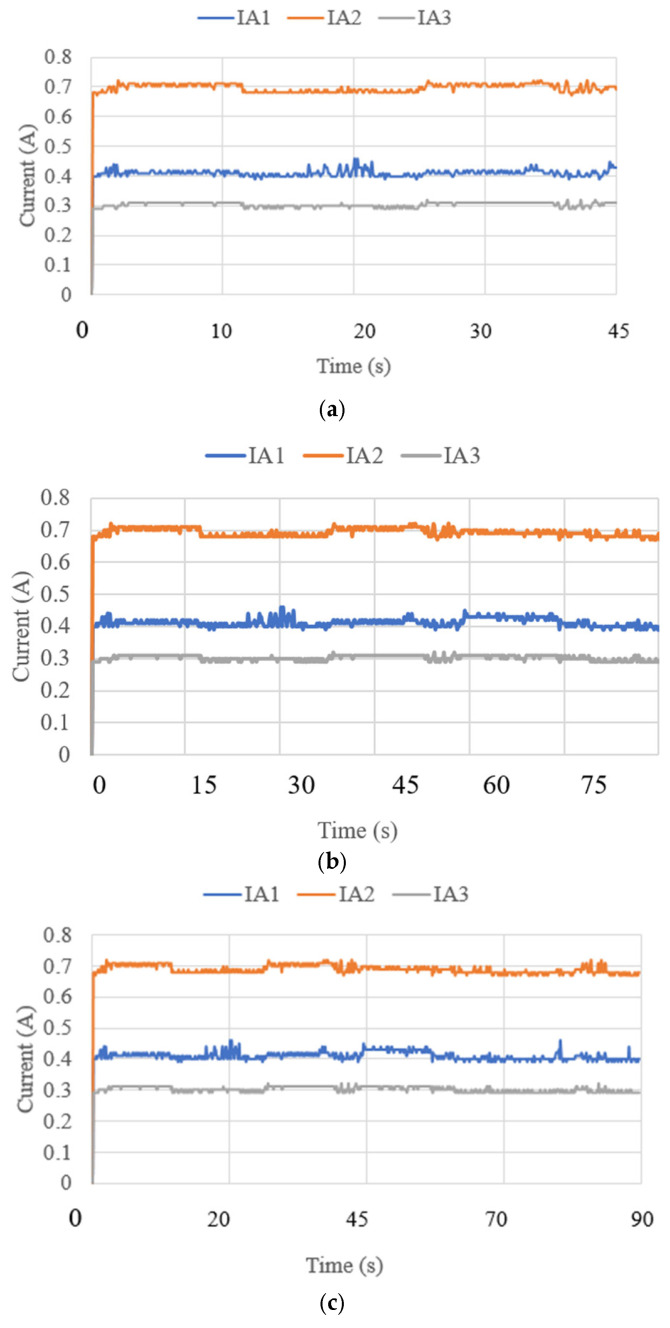
Current acquired from test mode 1 with the NeurADe model from Figure 9: (**a**) 45 s, (**b**) 75 s, and (**c**) 90 s.

**Figure 13:**
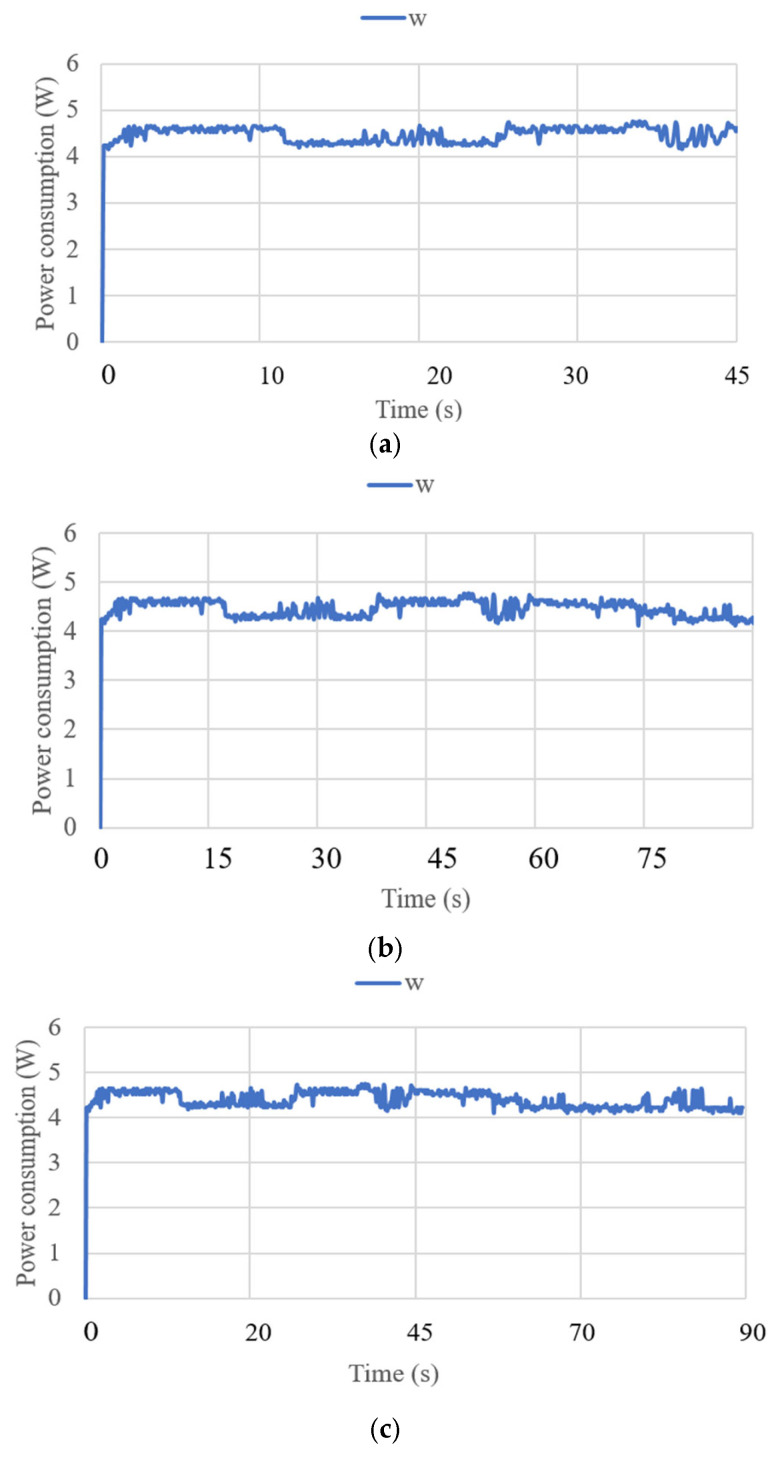
Power consumption was acquired from test mode 1 with NeurADe, as shown in Figure 9: (**a**) 45 s, (**b**) 75 s, and (**c**) 90 s.

**Figure 14:**
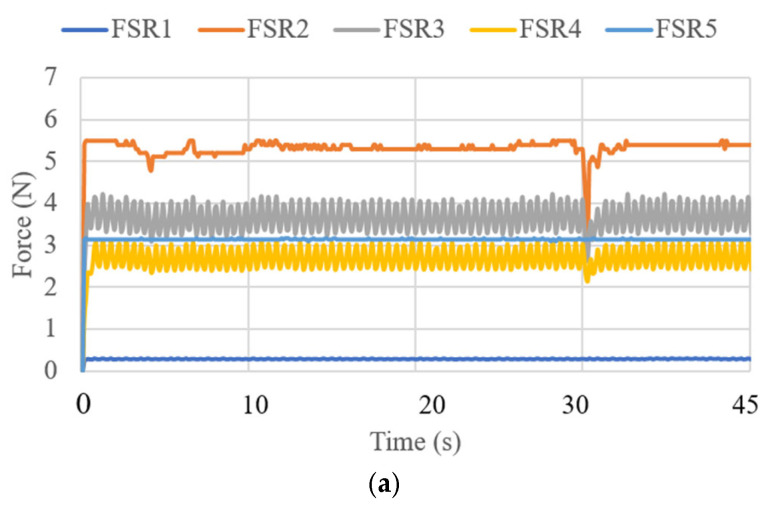

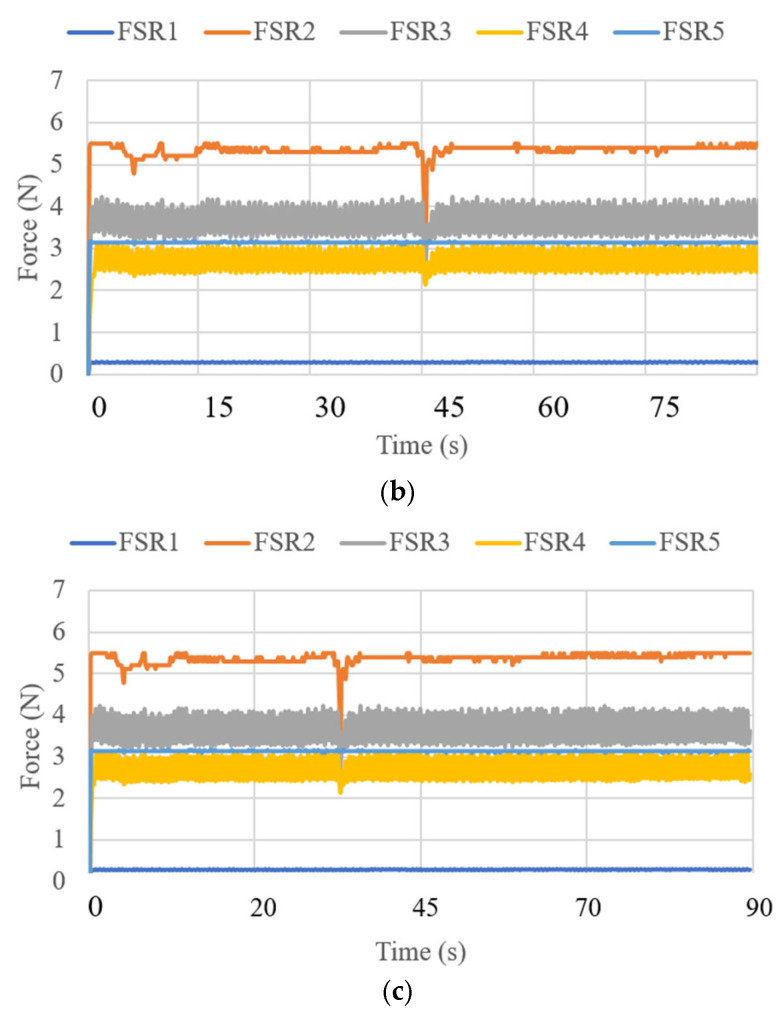
Force acquired from test mode 1 with the NeurADe model from Figure 9: (**a**) 45 s, (**b**) 75 s, and (**c**) 90 s.

The results of each test are summarized via accelerations, angles, current, power consumption, and forces. Figure 10 presents the performance of the device in test mode 1 in terms of accelerations. The acceleration in x had a decreased range from −3 to −5 m/s^2^. The y axis resulted in a range from 4 to 6 m/s^2^, and the z axis resulted in a range from 4 to 7 m/s^2^. The results were expected due to the device′s position in the cranium and the slow motion required for neurosurgery. The module of acceleration [34] was calculated as follows:

(1) $$a=\left|\sqrt{{a_{x}}^{2}+{a_{y}}^{2}+{a_{z}}^{2}}-g\right|,$$

where *g* is the gravity equal to 9.81 m/s^2^. The acceleration results are limited to 0.7 m/s^2^ and correspond to the small oscillations found during the data acquisition for the IMU. The angles acquired with the NeurADe in test mode 1 are shown in Figure 11. The pitch angles resulted in a range from 30 to 50°, and the roll angles ranged from −30 to 20°. The yaw angles were calculated from 30 to 50°. The results were as expected for the motion during neurosurgery, satisfying the requirements of the performance of the NeurADe. Figure 12 shows the current acquired from the linear actuators. The results of IA1, IA2, and IA3 are 0.4 A, 0.7 A, and 0.3 A, respectively. The current results show that the linear actuators operate following the movements indicated by the acceleration, and angles as illustrated in Figure 10 and Figure 11. The power consumption [34] is calculated as follows:

(2) $$W={I_{1}}^{2}V+{I_{2}}^{2}V+{I_{3}}^{2}V,$$

where V is the voltage of the supply current at 6 V, and I_i_ (i = 1, 2, 3) is the current of the three linear actuators. The primary power consumption was calculated within a range of 4 to 5 W. The expected power consumption values for the activation of the linear actuators during their movements over 45 s of operation are presented in Figure 13. Figure 14 displays the force obtained during test mode 1. The FSR sensor has a repeatability of ±6% [31]. For its configuration, a resistance of 47 KOhms and a voltage divider circuit were implemented. The calculation for each of the sensors was generated in the Arduino program. The force amount was from 0.5 N to 4 N in FSR1, FSR3, FSR 4, and FSR5. The force did not exceed the amount registered in [21] as a maximum force of 14 N to be applied to the screws. FSR2 is the force sensor at the end of the cannula, and for test mode 1, the detected force ranged from 3.5 to 5.5 N. The cannula was not stressed by any force, so the recorded force only reflects the moment the cannula touched the left parietal lobe.

**Figure 15:**
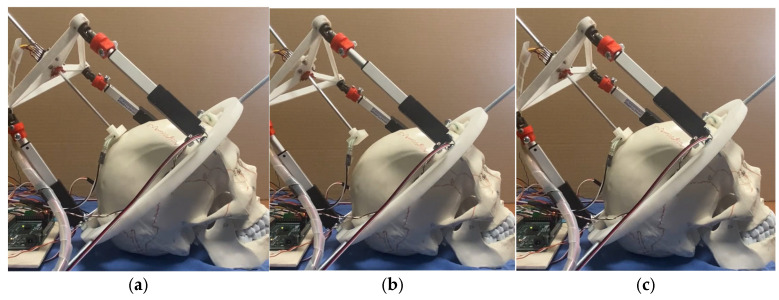
Performance of NeurADe in test mode 2: (**a**) start position, (**b**) performance to X2, and (**c**) final position.

**Figure 16:**
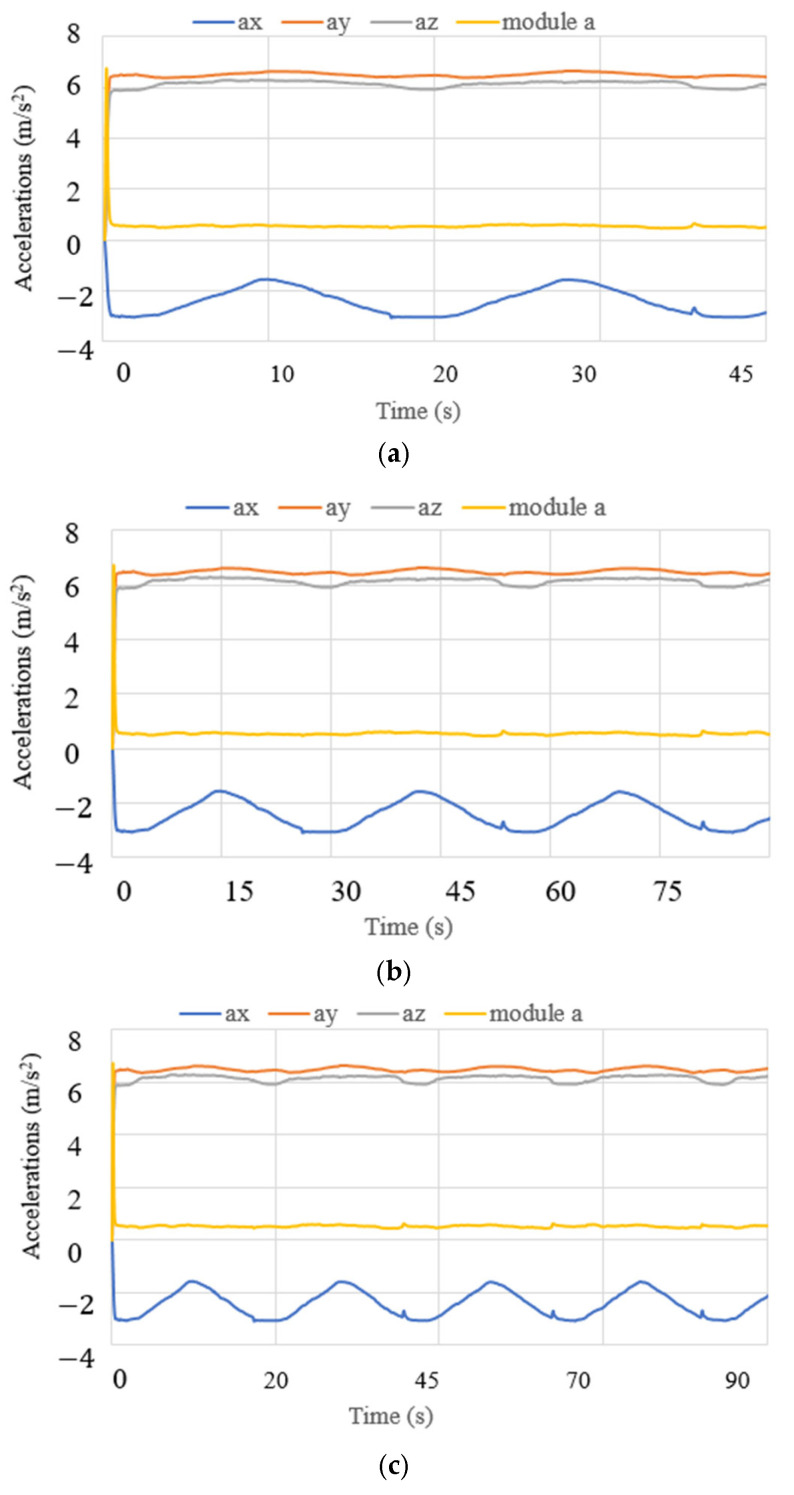
Acceleration acquired from test mode 2 with the NeurADe from Figure 15: (**a**) 45 s, (**b**) 75 s, and (**c**) 90 s.

**Figure 17:**
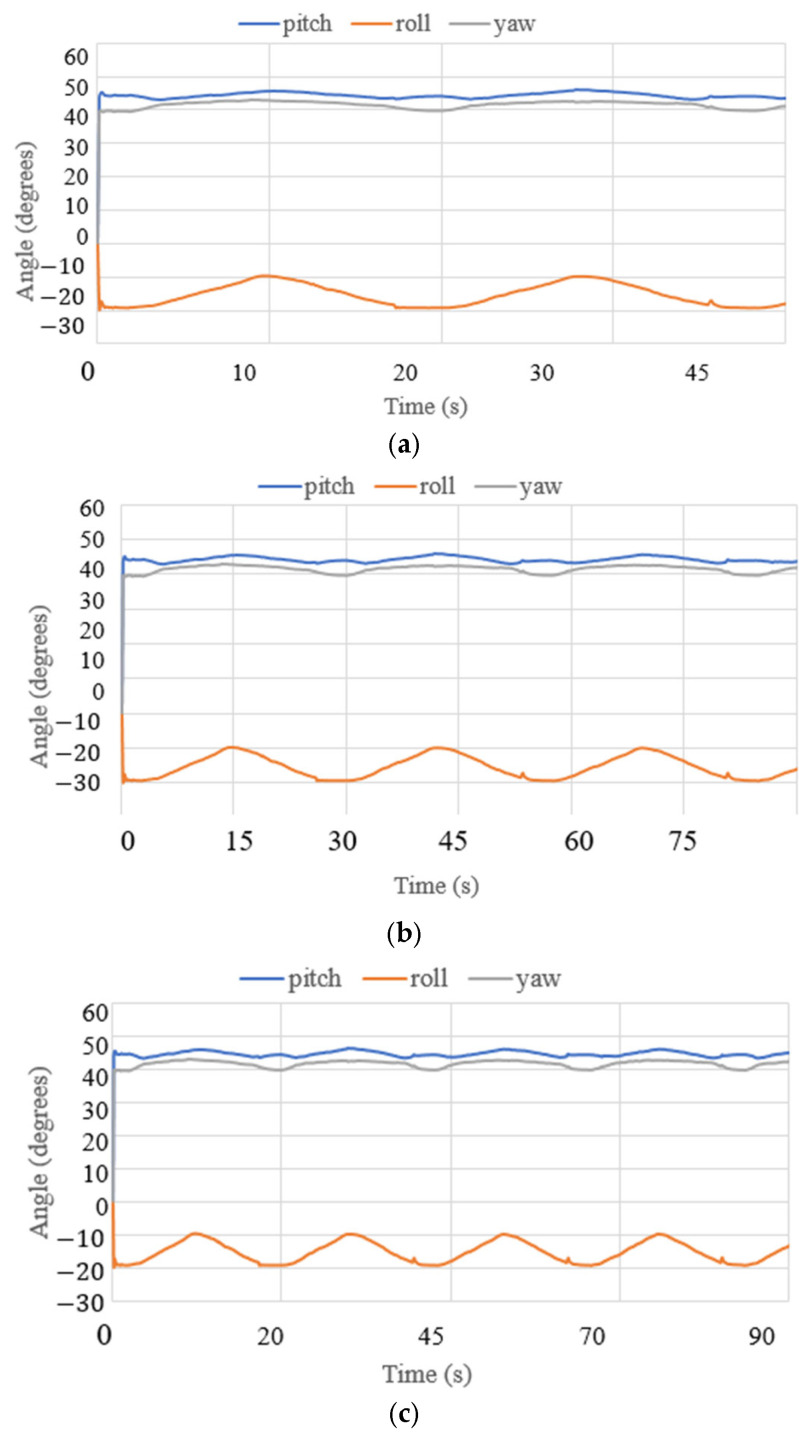
Angles acquired from test mode 2 with the NeurADe from Figure 15: (**a**) 45 s, (**b**) 75 s, and (**c**) 90 s.

**Figure 18:**
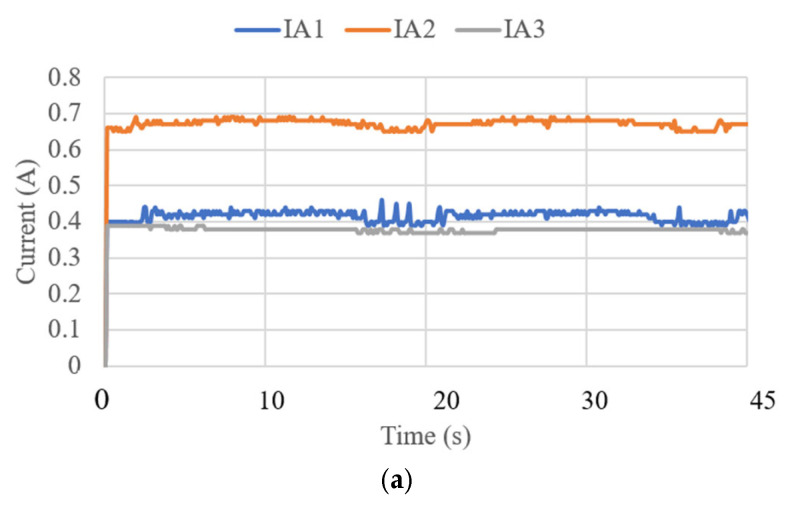

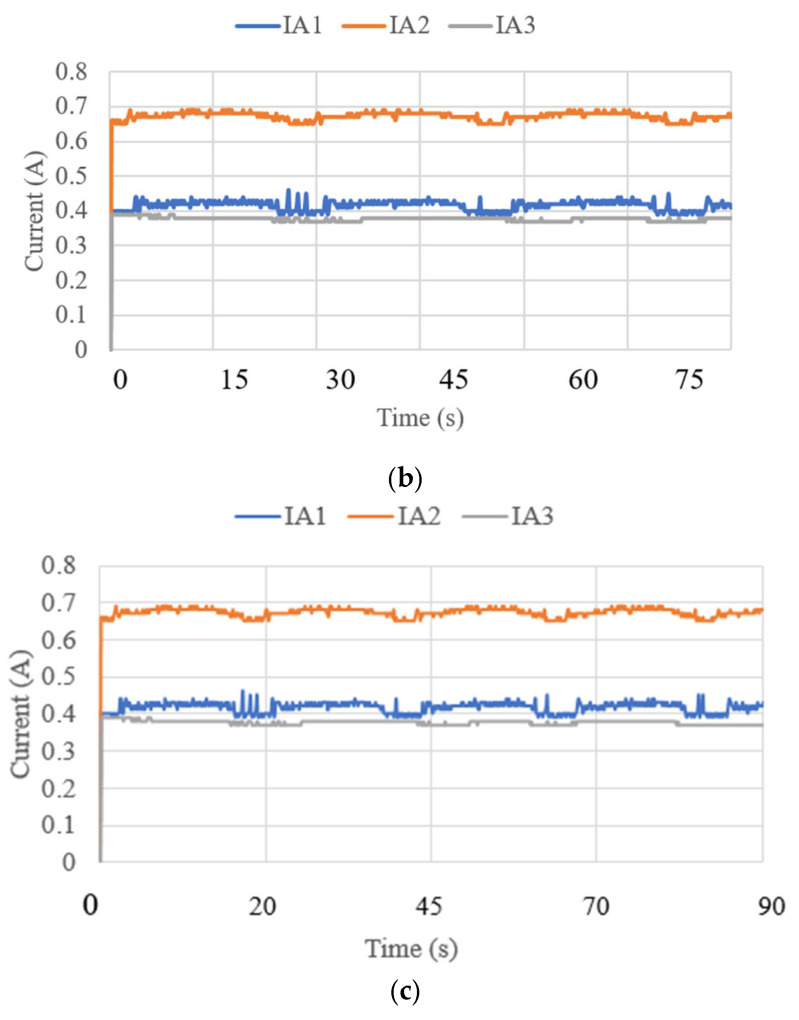
Current acquired from test mode 2 with the NeurADe from Figure 15: (**a**) 45 s, (**b**) 75 s, and (**c**) 90 s.

**Figure 19:**
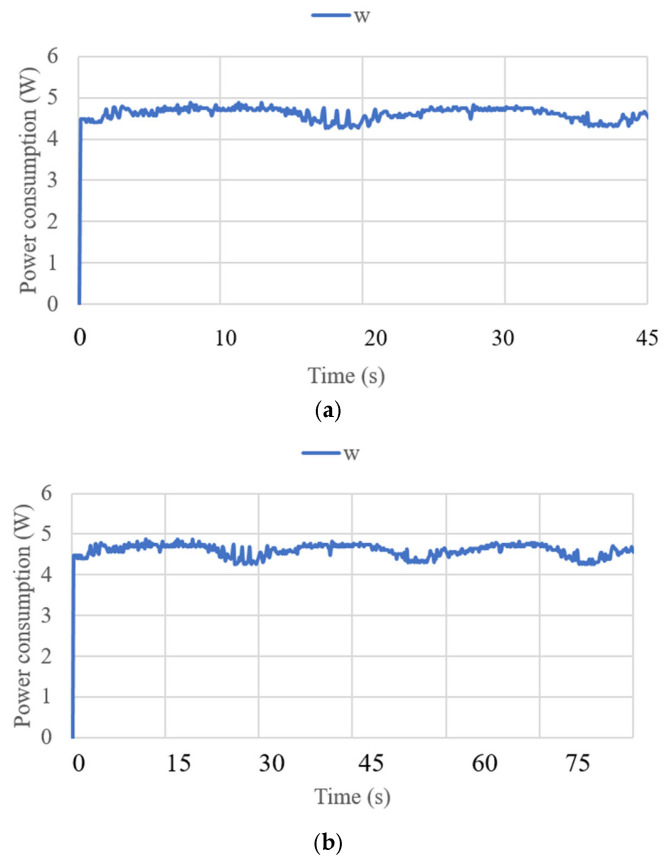

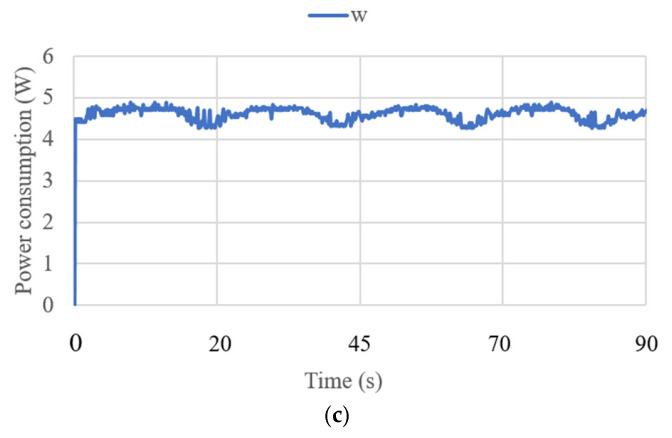
Power consumption was acquired from test mode 2 with NeurADe, as shown in Figure 15: (**a**) 45 s, (**b**) 75 s, and (**c**) 90 s.

**Figure 20:**
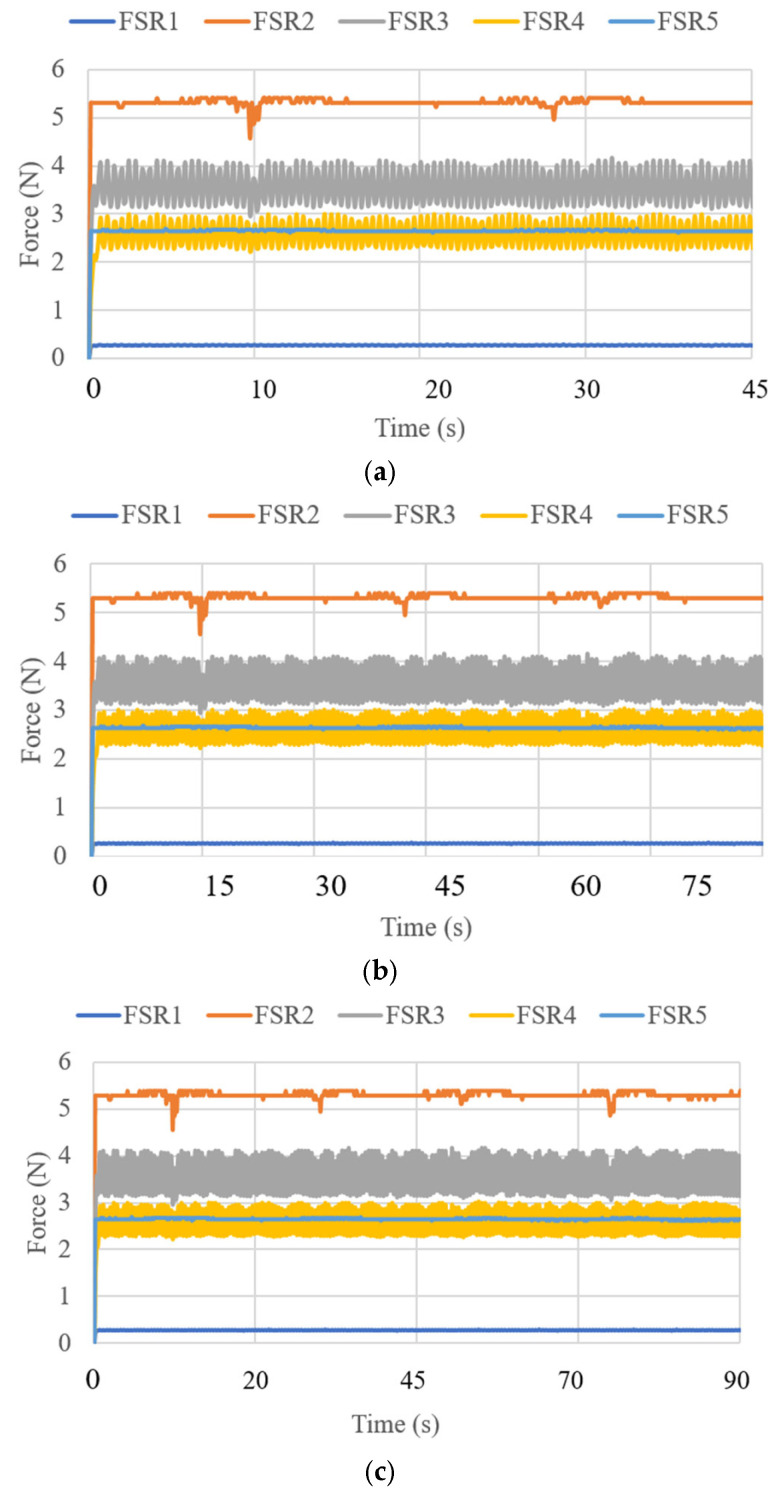
Force acquired from test mode 2 with the NeurADe from Figure 15: (**a**) 45 s, (**b**) 75 s, and (**c**) 90 s.

Test mode 2 was focused on the performance of NeurADe in the zone of the correct parietal lobe zone, as presented in Figure 15. The device′s operation started with the linear actuators at a stroke of 0 mm. LA1 and LA2 moved to their maximum positions with a stroke of 25 mm each. The results are presented in the same way as test mode 1’s results regarding accelerations, angles, current, power consumption, and force. The times were set at 45 s, 75 s, and 90 s for the experiment duration. Figure 16 shows the acquired data. The acceleration along the x axis had a range from −5 to −3 m/s^2^, the y axis acceleration ranged from 5 to 7 m/s^2^, and the z axis acceleration ranged from 4 to 7.5 m/s^2^. The obtained results are similar to those from test mode 1, where the acceleration module was calculated with equation 1 as 0.9 m/s^2^. The angles are reported in Figure 17. The roll angles had a range from −20° to −10°, the pitch angles ranged from 42° to 44°, and the yaw angles ranged from 39° to 41°. The computed angles presented the maximum and minimum values every 200 ms. The current of the linear actuators is shown in Figure 18. The current of the linear actuators ranged from 0.39 A to 0.65 A without exceeding the maximum permitted values. The power consumption is presented in Figure 19, as calculated with equation 2 for test mode 2, ranging from 4.2 W to 5 W. The power consumption of the device remained low, ensuring proper functionality. The force measured in test mode 2 is shown in Figure 20. The force generated at the contact points with the cranium ranged from 0.2 N to 4 N. As a result, in test mode 1, data from FSR3 and FSR4 were visualized with noise in the acquired signal. The FRS2 in the cannula detected a range from 4.5 N to 5.3 N.

## 4. Conclusions

This paper discusses the design and performance of the proposed neurosurgery-assisting device called a NeurADe. The design addresses the medical and technical requirements necessary for frame neurosurgery-assisting devices. Data acquired from an IMU, current sensors, and force sensors validate the performance of the device in terms of accelerations, angles, current, power consumption, and force. The NeurADe demonstrates the expected accelerations and angle performance that are required for positioning the device in the left and right parietal lobes. The current and power consumption data from the linear actuators indicate proper activation and motion during each test mode. Future work will be focused on testing the device with different trajectories and its application in human subjects.

## 5. Patents

“Plataforma para colocación de Electrodos de Estimulación Cerebral” Mexico, patent pending.

## Figures and Tables

**Table 1:** Experimental test modes with NeurADe.

Test Mode	Input	Output
1	Left parietal lobe X1	Accelerations, angles, currents, power consumption, and forces.
2	Right parietal lobe X2	Accelerations, angles, currents, power consumption, and forces.

## Data Availability

The original contributions presented in this study are included in the article. Further inquiries can be directed to the corresponding author(s).

## Abbreviations

NeurADe	Neurosurgery-Assisting Device
3-RPS	3- Revolute Prismatic Spherical Joint
DoF	Degrees of Freedom
